# Analysis of Apps With a Medication List Functionality for Older Adults With Heart Failure Using the Mobile App Rating Scale and the IMS Institute for Healthcare Informatics Functionality Score: Evaluation Study

**DOI:** 10.2196/30674

**Published:** 2021-11-02

**Authors:** Yohanca Maria Diaz-Skeete, David McQuaid, Adewale Samuel Akinosun, Idongesit Ekerete, Natacha Carragher, Lucia Carragher

**Affiliations:** 1 NetwellCASALA Advanced Research Centre Dundalk Institute of Technology Dundalk Ireland; 2 CCT College Dublin Dublin Ireland; 3 University of Highlands and Islands Inverness United Kingdom; 4 Ulster University Belfast United Kingdom; 5 National Drug and Alcohol Research Centre University of New South Wales Sydney Australia; 6 Alcohol, Drugs and Addictive Behaviours Department of Mental Health and Substance Use World Health Organization Geneva Switzerland

**Keywords:** mobile app, mHealth, medication app, heart failure, Mobile App Rating Scale

## Abstract

**Background:**

Managing the care of older adults with heart failure (HF) largely centers on medication management. Because of frequent medication or dosing changes, an app that supports these older adults in keeping an up-to-date list of medications could be advantageous. During the COVID-19 pandemic, HF outpatient consultations are taking place virtually or by telephone. An app with the capability to share a patient’s medication list with health care professionals before consultation could support clinical efficiency, for example, by reducing consultation time. However, the influence of apps on maintaining an up-to-date medication history for older adults with HF in Ireland remains largely unexplored.

**Objective:**

The aims of this review are twofold: to review apps with a medication list functionality and to assess the quality of the apps included in the review using the Mobile App Rating Scale (MARS) and the IMS Institute for Healthcare Informatics functionality scale.

**Methods:**

A systematic search of apps was conducted in June 2019 using the Google Play Store and iTunes App Store. The MARS was used independently by 4 researchers to assess the quality of the apps using an Android phone and an iPad. Apps were also evaluated using the IMS Institute for Healthcare Informatics functionality score.

**Results:**

Google Play and iTunes App store searches identified 483 potential apps (292 from Google Play and 191 from iTunes App stores). A total of 6 apps (3 across both stores) met the inclusion criteria. Of the 6 apps, 4 achieved an acceptable MARS score (3/5). The Medisafe app had the highest overall MARS score (4/5), and the Medication List & Medical Records app had the lowest overall score (2.5/5). On average, the apps had 8 functions based on the IMS functionality criteria (range 5-11). A total of 2 apps achieved the maximum score for number of features (11 features) according to the IMS Institute for Healthcare Informatics functionality score, and 2 scored the lowest (5 features). Peer-reviewed publications were identified for 3 of the apps.

**Conclusions:**

The quality of current apps with medication list functionality varies according to their technical aspects. Most of the apps reviewed have an acceptable MARS objective quality (ie, the overall quality of an app). However, subjective quality (ie, satisfaction with the apps) was poor. Only 3 apps are based on scientific evidence and have been tested previously. A total of 2 apps featured all the IMS Institute for Healthcare Informatics functionalities, and half did not provide clear instructions on how to enter medication data, did not display vital parameter data in an easy-to-understand format, and did not guide users on how or when to take their medication.

## Introduction

### Background

Managing the care of older adults with heart failure (HF) largely centers on symptom and medication management [1]. Medication management in patients with HF is challenging due to frequent medication or dosing changes [2,3] and polypharmacy, as some patients with HF typically take on average 10-25 tablets daily [4]. Polypharmacy is associated with poor adherence to pharmacological therapies, drug interactions, inappropriate drug prescriptions, and other adverse effects [5]. A recent report by the World Health Organization [6] argues that technology can improve patient experiences and medication adherence and enable patients to become active participants in medication reviews. Mobile apps offer the potential to augment care for patients with HF. Apps can potentially support older adults to find information on the medications (ie, drug interactions), track their medication, communicate with health care providers, keep a daily record of their blood pressure and weight measurements, and facilitate an accurate medication history. However, there is a dearth of literature on apps specifically to support medication history. An accurate medication list prevents adverse drug events [7], increases patients’ care outcomes, decreases hospitalization and mortality rates [8,9], and supports medication adherence for patients self-managing at home.

Given the complexity of HF self-care, assisting older adults in managing their own care at home is critical to the success of HF management. Emerging evidence suggests that mobile health (mHealth), particularly mobile technologies, can serve as a form of support for patients with HF and may enhance patient-provider collaboration for self-management [1,10]. By their nature, mobile devices, such as phones, are carried by people and, therefore, are always with them, offering opportunities beyond simple remote monitoring to assist with the management of care. In the current context of the COVID-19 pandemic, when the community (and especially older adults) is requested to maintain social distancing, the public health landscape is changing and mHealth has never been so important for treatment [10,11].

For older adults, social isolation and loneliness increase the risk of anxiety, depression symptoms, heart disease, reduction of activities of daily living, morbidity, and mortality [12,13]. Government recommendations to self-isolate during this pandemic have undoubtedly had a detrimental effect on older adults, including those that previously had wide social connections with the community and relatives [14]. Older adults who were previously attending outpatient appointments have seen their access restricted. In Ireland, the Health Service Executive website notes that all outpatient appointments are postponed until further notice [15]. Health care professionals (HCPs) working in outpatient clinics are seeing a reduced number of patients, with most consultations now taking place over the phone, bar medical emergencies. In Ireland, McGlynn [16] drew attention to the sharp decline in cardiac outpatient appointments during March to April 2020 (300,000 appointments) compared with the same period in 2019. Therefore, the need for new models of care in this changed environment to support older adults at home to alleviate their mental and physical burden, as well as provide medical care, is especially timely [10,11].

Across many countries, emerging evidence confirms the important role that mHealth can play in community care, especially during COVID-19. In the United States, there have been 10-fold web-based consultations in a few weeks [17], “...as big a transformation as any ever before in the history of US health care.” Similarly, Canada, South Africa, India, and the United Kingdom are conducting health care web-based consultations at an exponential rate [17]. In Ireland, the platform *Attend Anywhere*, endorsed by the Health Service Executive, is now widely available for HCPs to conduct web-based consultations [18]. However, the use of this platform is not even across Irish clinical settings (some HCPs are actively using it and others are not). Many outpatient services are consulting patients over the phone during the COVID-19 pandemic [19,20], except for patients with exacerbated symptoms.

The process of medication review over the phone is difficult and time-consuming [21]. HCPs have to listen attentively to the information the patient is conveying while lacking visual cues (ie, printed medication list or medication blisters provided by pharmacists). Instead, each patient has to spell each medication list over the phone, raising confusion over similarities of the medication name or dose, thus increasing the length of the consultation. An app sharing an up-to-date medication list with HCPs preconsultation could reduce medication and dosing errors, making the consultation process more efficient. A usability study of an app developed for HF self-management was conducted in Australia [22]. A total of 8 participants tested the app over a 14-day period and 6 of them assessed the app using the Mobile App Rating Scale (MARS; widely used to assess the quality of mobile apps). The app was found to be of acceptable quality and beneficial for HF self-management. Interestingly, the medication list feature on the app was considered by the patients to be beneficial; however, none of the patients used it during the 14 days. The authors suggested that participants found it difficult to incorporate the app into their self-care routine [22].

Emerging evidence suggests that apps can support patients by checking drug interactions, tracking medication intake, and facilitating an up-to-date list of medications. However, although some app interventions have explored HF self-management [23,24], app interventions focusing on older adults with HF maintaining an accurate medication list have been limited and largely unexplored. This paper aims to explore the benefits of apps with a medication list functionality, explore their role in the COVID-19 pandemic context, and assess their quality using the MARS and the IMS Institute for Healthcare Informatics functionality score.

### Objectives

The objectives of this review are two-fold: to review apps with a medication list functionality and to evaluate the quality of the apps included in the review with a validated scale. To assess the quality and functionality of the apps, 2 tools were used: the MARS tool and the IMS Institute for Healthcare Informatics functionality score. For the purpose of this paper, an app with a medication list functionality is an app that generates a comprehensive medication history, allowing the patient to email or share the list in real time with HCPs.

## Methods

### Overview

A systematic search of apps accessible in Ireland was conducted in June 2019 using the Google Play and iTunes App stores. The purpose of this search was to identify apps with a medication list functionality. The search term *medication list* was used to identify apps with a medication list functionality. The term *medication app* was excluded from the search as it identified apps with a different primary purpose (eg, medication alarm, medication tracker, medication reminder, apps providing educational information only, medical decision support systems for clinicians, medication adverse effect, pharmacy locator, and prescription refills). After the initial identification of apps containing a medical list function, the apps were tabulated. If the same app was available on different platforms (iOS or Android), both versions were retained for analysis as apps behave differently depending on the platform, as seen in previous work by Nicholas et al [25]. Inclusion and exclusion criteria (discussed next) were applied to each app to determine whether they should be retained for further analysis.

Apps that met the inclusion criteria were downloaded and evaluated by a team of 4 researchers. The MARS was used to assess the quality of the apps using an Android phone and an iPad. Apps were also evaluated using the IMS Institute for Healthcare Informatics functionality scores.

A Google Scholar search of the apps was conducted to identify apps included in this review that have been evaluated and published in peer-reviewed journals.

### Inclusion and Exclusion Criteria

Apps were included if they had a medication list function; if they were updated in the last 2 years; were free of charge, reflecting popular trends in app downloads [26]; were available in English; and had a strict privacy policy written on their website or app store. Although a strict privacy policy is not an ultimate standard, given the sensitive nature of health information, the presence of a transparent privacy policy on medication apps was deemed highly important [27].

Apps were excluded from evaluation if they were a game app, were not available in Ireland, focused solely on a particular medical condition (eg, asthma), were a mobile clinical decision support system, were designed primarily for self-care management of a condition (eg, chronic obstructive pulmonary disease or diabetes), were not available for patients to use at home, and had a barcode scanner that did not recognize medication used in Ireland.

### Data Extraction

The following information for each app with a medication list function was downloaded: developer, number of downloads of the app, last update, and description of the app in the app store. The apps were downloaded from the app store, and scientific support was evaluated by investigating their content. Scientific support provides information on the app’s evidence of validity, as apps that are not supported by evidence are associated with decrements in quality and safety [28]. For example, issues relating to patient confidentiality, inadequate content present in the app, and malfunctioning clinical decision-making apps could result in negative health outcomes for patients [29]. Apps that fulfilled the inclusion criteria were assessed using the MARS and IMS Institute for Healthcare Informatics functionality scoring criteria [30].

### Rating Tools

#### MARS Description

All apps were subjected to in-depth analysis and evaluation using the MARS. To the best of our knowledge, there are no published studies using the MARS to assess the quality of apps with a medication list functionality. The MARS was developed by a team of researchers at the University of Queensland, Australia, to provide a systematic means of assessing, classifying, and rating the quality of mHealth apps [31].

Within this framework, apps are rated according to 4 objective measures (engagement, functionality, aesthetics, and information quality) and one subjective measure (Table 1). More specifically, engagement involves determining whether the app is fun, interesting, customizable, interactive, and well-targeted to its audience. Functionality assesses whether the app is easy to learn, navigate, and flow logically. The esthetics category evaluates the graphic design, overall visual appeal, color scheme, and stylistic consistency of the app. Information quality involves evaluating whether the app contains high-quality information from a credible source. Subjective quality reflects user satisfaction, app endorsement, and continuity of use (ibid). A complete description of the MARS items and subscales can be found in Multimedia Appendix 1 [31].

**Table 1:** The Mobile App Rating Scale section scores, overall mean, subjective quality results, and mean total.

App name	Developer	Scores^a^					Overall mean^b^		Subjective quality results^c^
		Engage	Function	Aesthetics	Information				
Dosecast^d^	Montuno Software, LLC	3.3	3.4	3.3	3.1	3.3		2.5	
MyTherapy^d^	Smart patient GmbH	3.4	4.0	3.8	3.4	3.7		3.4	
Medication List & Medical Records^d^	LSD infotech	2.1	2.9	2.6	2.3	2.5		1.6	
Medisafe^d^	MediSafe	3.6	4.5	3.9	4.0	4.0		4.0	
MedList Pro^d^	Ramtin Software Solutions	3.2	3.1	2.8	2.7	3.0		1.5	
Dosecast^e^	Montuno Software, LLC	3.1	3.2	3.2	2.8	3.1		2.2	
My Therapy^e^	Smart patient GmbH	3.4	3.9	3.6	3.2	3.5		2.9	
Medisafe^e^	MediSafe Inc	3.6	4.5	3.9	4.0	4.0		3.8	
Pill Reminder^e^	Sergio Licea	3.5	4.1	3.9	3.1	3.7		3.6	

^a^Engagement: mean 3.24 (SD 0.461); functionality: mean 3.73 (SD 0.602); aesthetics: mean 3.42 (SD 0.506); information: mean 3.17 (SD 0.565).

^b^Overall: mean 3.4 (SD 0.496).

^c^Subjective: mean 2.82 (SD 0.945).

^d^Android platform.

^e^iTunes platform.

The apps were independently reviewed by 4 reviewers using a five-point scale (1=inadequate, 2=poor, 3=acceptable, 4=good, and 5=excellent), as shown in Multimedia Appendix 2. Scores for each category were obtained by calculating the mean of the ratings for each subscale according to the 5 measures described above. The total score for each app was determined using the average of the 4 objective measures. The overall mean app quality total score and the total score for the subjective measure (subjective quality, worth recommending, repeat use of the app, and overall satisfaction) were also calculated.

The reviewers carefully read the MARS instructions, independently reviewed the apps, and provided a rationale for their ratings. Subsequently, they compared the results and reached a consensus on each of the ratings for each of the MARS subscales [31]. Before rating the included apps, each reviewer rated 2 randomly selected apps for training purposes (from those apps that were excluded from the review). The results were discussed to ensure that all reviewers had an understanding of the MARS items and the rating process.

#### IMS Institute for Healthcare Informatics Functionality Score Description

To complement the MARS quality assessment, another tool was used to independently evaluate app functionalities [30]. This evaluation focused on the scope of functions and the potential role that each functionality plays in supporting self-management for patients with HF.

Unlike MARS, this tool only assesses objective quality and has been used previously to evaluate app capabilities [32,33]. The functionality score consists of 7 functionality criteria and 4 functional subcategories. The complete structure of the IMS Institute for Healthcare Informatics functionality scoring criteria can be found in Multimedia Appendix 3 [30]. If a function was present, it was coded as 1; otherwise, it was coded as 0. Functionality scores ranging from 0 to 11 were generated for each app.

## Results

### Overview

Google Play and iTunes App stores searches identified 483 potential apps (292 Google Play stores and 191 iTunes App stores), the app selection process for both app stores can be seen in Multimedia Appendices 4 and 5. A total of 6 apps (3 across both stores) met the inclusion criteria. Out of the 6 apps reviewed, 4 achieved an acceptable quality score (MARS score 3/5), one achieved a good quality score (4/5), and one had a poor quality score (2.5/5). The median overall MARS score was 3.5/5, ranging from 2.5/5 to 4/5 (mean 3.4, SD 0.49). As stated earlier, the apps are rated according to 4 objective measures: engagement, functionality, aesthetics, and information. The functionality dimension mean score achieved the highest score (3.7), whereas the mean score for the information and engagement dimensions was the lowest (3.2). The total mean subjective MARS score (2.8/5) was lower than the total mean objective MARS score (3.4/5).

On average, the apps had 8 functions based on the IMS criteria (range 5-11). Two apps achieved the highest IMS functionality criteria score (11 functions), whereas 2 apps achieved the lowest score (5 functions). All functions (n=11) are listed in Table 2. All apps had collecting, sharing, and recording functionality. However, half of the apps did not provide clear instructions on how to enter medication data, did not display vital parameter data in an easy-to-understand format, and did not guide or provide users with advice on how or when to take their medication. Only 2 apps allowed users to communicate with HCPs, family, and friends in real time.

**Table 2:** IMS Institute for Healthcare Informatics functionality score results.

IMS functionality scoring criteria	Dosecast (Google Play and iTunes)	My Therapy (Google Play and iTunes)	Medication List & Medical Records (Google Play)	Medisafe (Google Play and iTunes)	Pill Reminder (iTunes)	MedList Pro (Google Play)
Inform		✓^a^		✓		
Instruct		✓		✓	✓	
Record	✓	✓	✓	✓	✓	✓
Collect data	✓	✓	✓	✓	✓	✓
Share data	✓	✓	✓	✓	✓	✓
Evaluate data		✓	✓	✓	✓	
Intervene	✓	✓		✓	✓	✓
Display		✓	✓	✓		
Guide		✓		✓		✓
Remind or alert	✓	✓		✓	✓	✓
Communicate		✓		✓		
Total functions present	5	11	5	11	7	6

^a^✓: Function present in the app.

### Quality Assessment Using the MARS

Four out of the six apps achieved acceptable quality, suggesting that most apps are of acceptable quality. None of the apps presented any major technical issues during the review, and all were updated in the last 2 years. Features included in most apps reviewed were medication reminders, medication history logs with the ability to share a medication report with others, vital parameter tracking, and syncing the account to other devices.

Only one app (Medisafe) achieved the highest objective and subjective overall MARS scores. One of the distinctive features of this app was the ability to educate users on how and when to take their medication, drug-drug interaction information, and medication side effects. This information was presented in videos using a clear and concise language and text format. In addition, there is evidence of effectiveness, as the Medisafe app has been previously tested in 2 randomized controlled trials [34,35], as a medication adherence tool using scheduled reminders [36] and as a medication reminder related to patients’ intention to use the app [37].

The overall subjective quality dimension was significantly lower (2.8/5) than the overall objective mean of all apps (3.4/5). Only one app (Medication List & Medical Records) achieved a lower objective overall score (2.5/5) than the subjective overall score. The subjective quality represented the opinion of the user on the level of satisfaction with the app, willingness to pay for it, and the extent to which the user will recommend it to others. It appears that the apps reviewed, to a certain extent, are well designed, as most apps achieved an acceptable objective quality. However, the subjective quality was poor, as the reviewers provided lower scores on continuity of use and willingness to recommend the apps to others.

The functionality dimension mean score achieved the highest score (3.7), indicating a trend toward higher responses in a timely manner, intuitiveness and ease of use, and navigation across all apps. However, the mean score for the information and engagement dimensions was the lowest (3.2). The information dimension represents the quality and quantity of information present in the app and the way this information is provided, for example, through the use of different formats, such as videos, text, or graphs. The quality of the information on the apps reviewed varied, as some provided good quality medication information; in others, there was a very poor level of information or none present at all.

None of the apps included in the review performed very well in the engagement dimension (if the app was fun or interesting to the user). Medication apps as a rule are not fun and entertaining; however, most apps allow interactivity (ability to input information and prompting) and customization (sending notifications and setting up medication reminders). Another category in engagement, the target group, evaluated if the app content (ie, visual information, language, and design) was appropriate for the user. Most patients diagnosed with HF are older adults. The apps reviewed were not specifically designed for use by older adults and did not have an age-friendly interface. An example of an age-friendly interface is when an app facilitates older adults to enlarge the font size of the screen if required. Avoiding information overload and providing detailed instructions on how to use the app are also age-friendly interface examples [38]. Three of the apps reviewed (Dosecast, Medication List & Medical Records, and MedList Pro) did not provide clear instructions on how to use the app or how to input medication. App developers should consult with and take older adults’ views, needs, and preferences into consideration to make apps more age-friendly and increase usability [38].

### IMS Institute for Healthcare Informatics Functionality Score Evaluation and Implications for Patients With HF

#### Inform

Only 2 apps (Medisafe and MyTherapy) communicated effectively to users, offering an educational component about medication and the medical condition associated with each medication. In addition, both apps informed users about the medication they are taking and possible drug-to-drug interactions. Due to frequent medication or dosing changes in patients with HF, this is a critical function. Medication adherence and continuous education on the condition and symptom management are vital to reduce rehospitalization, illness progression, and exacerbation of symptoms in patients with HF [39].

#### Instruct

Some of the apps reviewed (Dosecast, Medication List & Medical Records, and MedList Pro) were not intuitive and did not provide clear instructions on how to enter medication strength, time of the day, route, and setting up medication reminders. In other studies, the level of detailed instructions varied. Providing clear instructions on the use of an app is vital for older adults with HF. Patients with HF are predominantly patients aged ≥ 65 years [40], and old age has been cited as a barrier to app use and uptake [41]. Therefore, app designers should consider the needs of older adults using apps and include basic usability advice and easy-to-understand content [38,42].

#### Record

All apps allowed users to record their medication and a history of use. Most apps also had the capability to record vital parameters. For patients with HF, blood pressure and weight measurements are useful for monitoring the progress of their illness and identifying when symptoms exacerbate. One of the main goals of HF care is to avoid rehospitalization and major adverse cardiac events [32]. In particular, one app offered the possibility of tracking mental well-being, another key area that needs particular attention. For individuals diagnosed with HF, depression and anxiety are common, leading to rehospitalization, poorer quality of life, and increased morbidity and mortality [43].

#### Evaluate Data

Four apps (MyTherapy, Medication List & Medical Records, Medisafe, and Pill Reminder) allowed data entered into the app to be analyzed and evaluated by the user, a relative, or an HCP. This functionality allows for information to be shared easily and in a timely manner for HCPs to evaluate it and act accordingly. For example, relatives and clinicians can evaluate whether a person is adhering to medication.

#### Intervene

Five out of the six apps (except Medication List & Medical Records) had the capability to recommend the user, a relative, or a medical practitioner to intervene based on the data collected. Building from the example provided in “evaluate data,” once the health information is evaluated, an intervention can be put in place. Examples of interventions for patients with HF could be to reduce or increase the medication dose, reduce fluid intake, and attend the clinic or emergency department on the day. Other examples are the ability of the app to communicate effectively to provide a positive intervention, that is, reminders to refill their medication or a suggestion to engage in physical activity to achieve their daily activity goal.

#### Display

Apps with a cluttered or bland display do not engage users and are less likely to offer a positive user experience. In 3 of the apps reviewed (MyTherapy, Medication List & Medical Records, and Medisafe), the data were displayed in a clear and colorful graphical representation format. This function could potentially be effective for patients with HF as they can easily understand health reports, that is, medication, weight, and blood pressure [22]. Consequently, health reports will highlight behavioral changes, for example, to adhere to the medication prescribed, reduce fluid intake, or ring the clinic regarding weight gain.

#### Guide

Half of the apps (Dosecast, Medication List & Medical Records, and Pill Reminder) did not provide comprehensive guidance or training about the correct administration of medication or advice on the time of day that the medication should be administered, for example, before or after a meal. HF medication management is complex, and continuous education and advice on regular medication use is vital for HF self-management [32].

#### Reminder or Alert

All apps issued an alert to remind users to take their medication and most allowed the users to tick off their medication once they take it or record it as a missed dose. Most apps also had the capability of reminding users of upcoming medical appointments. Apps with a reminder function improved medication adherence and enhanced complex medication management in patients with HF [32]. One of the apps, Medisafe, alerted users and their relatives of drug-drug interactions and of missed medication doses.

#### Communicate

This function offers the option to communicate with HCPs or with an online support group in real time. The Medisafe app offers users the possibility to communicate with unlimited Medfriends supporters (relatives, friends, or caregivers). Another app, MyTherapy, provides HCPs with an overview of patients’ data to plan for their treatment between visits through the web dashboard function.

### Google Scholar Search

A search was conducted to identify the apps included in this review that have been evaluated and published in peer-reviewed journals. Out of all the apps reviewed, 3 apps were identified in the search: (1) Dosecast, (2) MyTherapy, and (3) Medisafe app.

## Discussion

### Principal Findings

To our knowledge, this is the first study to assess the quality of apps with a medication list functionality using the MARS and the IMS Institute for Healthcare Informatics functionality scale available to Irish consumers. The most common functionalities found in the apps reviewed were medication reminders, medication history logs, and the ability to share medication reports with others, vital parameter tracking, and syncing the account to other devices. However, half did not provide clear instructions on how to enter medication data, did not display vital parameter data in an easy-to-understand format, and did not guide users on how or when to take their medication.

App users prefer apps that are effective, useful, and easy to use [44]. From the apps reviewed, the Medisafe app achieved the highest objective and subjective overall MARS score and the highest IMS Institute for Healthcare Informatics functionality score. One of the distinctive features of this app is the ability to educate users on how and when to take their medication, drug-drug interaction information, and medication side effects. This information was presented in videos using a clear and concise language and text format. The app was found to be very intuitive and had an age-friendly interface.

The quality and efficacy of health apps is another important factor to be considered by users, as they provide positive user experiences and a higher uptake [45]. Most of the apps included in this review had acceptable quality. However, for users, it is not easy to determine the quality, performance, and trustworthiness of apps. The number of apps available in app stores has been growing exponentially in the last decade [30,46] impacting the user’s ability to distinguish app quality and performance. Therefore, a reliable and easy-to-use tool to facilitate this process is warranted [47].

The use of mHealth apps is growing at an exponential rate, but there are questions about their efficacy. One of the methods to check the evidence of efficacy is to conduct a search for peer-reviewed academic evidence. For the purpose of this study, a search was conducted for each app in Google Scholar to identify any published peer-reviewed articles. Three of the reviewed apps were identified in the search. The Medisafe app has been previously tested in 2 randomized controlled trials: (1) a medication adherence study [34] and (2) a medication adherence and blood pressure control study [35]. The MyTherapy app was tested in a study [48] as a medication tracker and reminder, whereas the Dosecast app was included in the IMS Institute for Healthcare Informatics review [30], a MARS review [49], and on a feasibility and acceptability medication adherence experimental trial [50]. However, many widely used apps have not yet been scientifically tested. Therefore, there is a need for more apps to be tested scientifically and the outcomes to be disseminated to inform the mHealth research community [22,51].

The mHealth research community has been actively searching for self-management solutions to support older adults shielding at home during the COVID-19 pandemic. One of their focus areas is to support self-management and medication adherence [52]. Apps with a medication reminder functionality may play a potentially important role in promoting greater self-management of vulnerable older adults living at home. In Ireland, as per March 2021, older adults are shielded, and movement is restricted to a 5 km radius. The monotonous routine makes each day very similar to the previous one, and daily routines, such as taking medication at scheduled times might become easy to forget. All apps reviewed, with the exception of one, have the capability of reminding users to take their medication. Medication reminder apps have been found to be effective and to increase medication adherence [48,49,53-55], even for patients with HF [56]. Another app feature that might be of benefit during the COVID-19 pandemic is the ability of older adults with HF to communicate with their medical team or relatives via the app. As mentioned earlier, in Ireland, the number of in-person HF outpatient consultations has considerably decreased by 2020 [15,16]. As of March 2021, the probability of contracting the virus for health care personnel remains high [57], and patients with HF are considered to be one of the most vulnerable groups [58].

Finally, this paper identified a small number of apps that could be suitable for patients with HF sharing their medication list with HCPs before consultation. Owing to the sensitive nature of health care data and in an era where General Data Protection Regulation (GDPR) legislation considerations are increasingly important, a strict data policy was a criterion for selection. For example, one of the most frequent concerns app users have is how their health data are processed. Under GDPR legislation, app users are encouraged to ask and obtain information in relation to data security and data processing [59]. However, after the introduction of the GDPR in 2018, no specific guidance on privacy has been developed for apps widely available to consumers [60]. Therefore, the need for transparent and easy-to-understand strict privacy policies in health apps should be mandatory if consumers are expected to download and use mHealth apps [60,61] and if clinicians are expected to recommend apps to their patients [62].

### Conclusions

The quality of current apps with a medication list functionality varies according to their technical aspects. Most of the reviewed apps have acceptable MARS objective quality. However, the subjective quality or satisfaction with the apps was poor. The objective quality assesses whether an app is interesting, easy to navigate, and overall visual appeal, among other characteristics. Subjective quality reflects user satisfaction, app endorsement, and continuity of use. Only 3 apps are based on scientific evidence and have been tested previously. Two apps featured all the IMS Institute for Healthcare Informatics functionalities and half did not provide clear instructions on how to enter medication data, did not display vital parameter data in an easy-to-understand format, and did not guide users on how or when to take their medication.

To our knowledge, this is the first study to use the MARS to assess the quality of apps with a medication list functionality available in the Irish app stores. The need for an app to support older adults with HF to maintain an accurate medication list is warranted. We recommend that app developers display either in the app or on the website, a transparent and easy-to-understand privacy policy to increase patients’ and HCPs’ trust and use.

### Limitations

One of the limitations of this review is the limited number of apps, as only those with a strict privacy policy written on their website or app store were included. Furthermore, due to the fast pace of app development, it is possible that by the time this paper is published, there may be new apps with a medication list functionality available to Irish consumers.

## Appendix

Multimedia Appendix 1 Mobile App Rating Scale items and subscales.

Multimedia Appendix 2 Mobile App Rating Scale ratings of included apps.

Multimedia Appendix 3 IMS Institute for Healthcare Informatics functionality scoring.

Multimedia Appendix 4 App selection process (Google Play app store).

Multimedia Appendix 5 App selection process (Apple app store).

## Abbreviations

GDPR: General Data Protection Regulation
HCP: health care professional
HF: heart failure
MARS: Mobile App Rating Scale
mHealth: mobile health
