# Supplementary material for: Analysis of Apps With a Medication List Functionality for Older Adults With Heart Failure Using the Mobile App Rating Scale and the IMS Institute for Healthcare Informatics Functionality Score: Evaluation Study
Source: JMIR Mhealth Uhealth. 2021 Nov 2;9(11):e30674. doi: 10.2196/30674 (PMC8596242; doi:10.2196/30674)
Supplement: Multimedia Appendix 1 [file mhealth_v9i11e30674_app1.docx]

Multimedia appendix 1: MARS items and subscales.

| Quality scoring criteria | Subscales |
| --- | --- |
| 1. Engagement | - 1. Entertainment   2. Interest   3. Customisation   4. Interactivity   5. Target group |
| 1. Functionality | - 1. Performance   2.2 Ease of use   - 1. Navigation   2.4 Gestural design |
| 1. Aesthetics | - 1. Layout   2. Graphics   3. Visual appeal |
| 1. Information | - 1. Accuracy of app description in app store   2. Goals of app   3. Quality of information   4.4 Quantity of information   - 1. Visual information   2. Credibility – source specified in app store or app itself   3. Evidence base – has the app been trialled / tested |
| 1. Subjective quality | - 1. Would you recommend this app to other people?   2. How many times do you think you would use this app in the next 12 months?   3. Would you pay for this app?   4. What is your overall star rating for this app? |

Source: Stoyanov SR, Hides L, Kavanagh DJ, Zelenko O, Tjondronegoro D, Mani M. Mobile app rating scale: a new tool for assessing the quality of health mobile apps. JMIR Mhealth Uhealth. 2015;3(1):e27

URL: http://mhealth.jmir.org/2015/1/e27/ doi:10.2196/mhealth.3422 PMID:25760773
This is an open-access article distributed under the terms of the Creative Commons Attribution License (http://creativecommons.org/licenses/by/2.0/)
