# Supplementary material for: Analysis of Apps With a Medication List Functionality for Older Adults With Heart Failure Using the Mobile App Rating Scale and the IMS Institute for Healthcare Informatics Functionality Score: Evaluation Study
Source: JMIR Mhealth Uhealth. 2021 Nov 2;9(11):e30674. doi: 10.2196/30674 (PMC8596242; doi:10.2196/30674)
Supplement: Multimedia Appendix 2 [file mhealth_v9i11e30674_app2.docx]

Multimedia appendix 2: mobile application rating scale (MARS) ratings of included apps.

|  | Engagement | Functionality | Aesthetics | Information | Subjective quality |
| --- | --- | --- | --- | --- | --- |
| Dosecast (Google Play) |  |  |  |  |  |
| Rater 1 | 2.4 | 3.5 | 2.7 | 2.5 | 2 |
| Rater 2 | 3.4 | 3.3 | 3 | 2.9 | 2.8 |
| Rater 3 | 3.4 | 2 | 3.3 | 2.3 | 2.5 |
| Rater 4 | 3.8 | 4.7 | 4.3 | 4.6 | 2.8 |
|  |  |  |  |  |  |
| Medisafe (Google Play) |  |  |  |  |  |
| Rater 1 | 3 | 4.5 | 4 | 4.3 | 3.3 |
| Rater 2 | 4 | 4.8 | 4 | 4 | 4.3 |
| Rater 3 | 3.6 | 3.8 | 3.3 | 3.6 | 3.3 |
| Rater 4 | 3.8 | 5 | 4.3 | 4.1 | 5 |
|  |  |  |  |  |  |
| MyTherapy (Google Play) |  |  |  |  |  |
| Rater 1 | 3.2 | 5 | 4 | 3.8 | 3.3 |
| Rater 2 | 4 | 2.3 | 3.3 | 2.7 | 2.8 |
| Rater 3 | 3 | 3.5 | 3.3 | 3.1 | 3 |
| Rater 4 | 3.4 | 5 | 4.3 | 4 | 4.5 |
|  |  |  |  |  |  |
| Medication list and medical records (Google Play) |  |  |  |  |  |
| Rater 1 | 2.4 | 3.3 | 3 | 2.3 | 2.3 |
| Rater 2 | 1.4 | 2 | 2 | 1.8 | 1.3 |
| Rater 3 | 1.4 | 2 | 2 | 1.8 | 1 |
| Rater 4 | 3 | 4.3 | 3.3 | 3.2 | 1.8 |
|  |  |  |  |  |  |
| MedList Pro (Google Play) |  |  |  |  |  |
| Rater 1 | 2 | 2.8 | 2 | 2 | 1.3 |
| Rater 2 | 3.4 | 2.3 | 2.3 | 2.3 | 1 |
| Rayer 3 | 3.4 | 2.3 | 2.3 | 2.3 | 1 |
| Rater 4 | 4 | 4.8 | 4.3 | 4 | 2.5 |
|  |  |  |  |  |  |
| Dosecast (iTunes) |  |  |  |  |  |
| Rater 1 | 2.4 | 3.5 | 2.7 | 2.5 | 2 |
| Rater 2 | 3.4 | 3.3 | 3 | 2.9 | 2.8 |
| Rater 3 | 3.4 | 2 | 3.3 | 2.3 | 2.5 |
| Rater 4 | 3 | 3.8 | 3.7 | 3.3 | 1.5 |
|  |  |  |  |  |  |
| Medisafe (iTunes) |  |  |  |  |  |
| Rater1 | 3 | 4.5 | 4 | 4.3 | 3.3 |
| Rater 2 | 4 | 4.6 | 4 | 4 | 4.3 |
| Rater 3 | 3.6 | 3.8 | 3.3 | 3.6 | 3.3 |
| Rater 4 | 3.8 | 5 | 4.3 | 4.1 | 4.3 |
|  |  |  |  |  |  |
| MyTherapy (iTunes) |  |  |  |  |  |
| Rater 1 | 3.2 | 5 | 4 | 3.8 | 3.3 |
| Rater 2 | 4 | 2.5 | 3.3 | 2.7 | 2.8 |
| Rater 3 | 3 | 3.5 | 3.3 | 3.1 | 3 |
| Rater 4 | 3.2 | 4.5 | 3.7 | 3.3 | 2.3 |
|  |  |  |  |  |  |
| Pill Reminder (iTunes) |  |  |  |  |  |
| Rtaer 1 | 3.2 | 5 | 4.7 | 3.3 | 4 |
| Rater 2 | 3 | 3.5 | 3.3 | 2.5 | 2 |
| Rater 3 | 3.6 | 3 | 3 | 2.4 | 3.5 |
| Rater 4 | 4.2 | 5 | 4.7 | 4.3 | 5 |
|  |  |  |  |  |  |
