# Supplementary material for: Analysis of Apps With a Medication List Functionality for Older Adults With Heart Failure Using the Mobile App Rating Scale and the IMS Institute for Healthcare Informatics Functionality Score: Evaluation Study
Source: JMIR Mhealth Uhealth. 2021 Nov 2;9(11):e30674. doi: 10.2196/30674 (PMC8596242; doi:10.2196/30674)
Supplement: Multimedia Appendix 3 [file mhealth_v9i11e30674_app3.docx]

Multimedia appendix 3: IMS institute for healthcare informatics functionality score.

| ***Functionality scoring criteria*** | ***Description*** |
| --- | --- |
| ***Inform*** | Provide information in a variety of formats (text, photo, video) |
| ***Instruct*** | Provide instructions to the user |
| ***Record***  ***Collect data***  ***Share data***  ***Evaluate data***  ***Intervene*** | Capture user entered data |
|  | Able to enter and store health data on individual phone |
|  | Able to transmit health data |
|  | Able to evaluate the entered health data by patient and provider, provider and administrator, or patient and caregiver |
|  | Able to send alerts based on the data collected or propose behavioural intervention or changes |
| ***Display*** | Graphically display user entered data/output user entered data |
| ***Guide*** | Provide guidance based on user entered information, and may further offer a diagnosis, or recommend a consultation with a physician/a course of treatment |
| ***Remind/Alert*** | Provide reminders to the user |
| ***Communicate*** | Provide communication with healthcare professional/patients and/or provide links to social networks |
|  |  |

Source: Aitken M, Gauntlett C. Patient apps for improved healthcare: from novelty to mainstream. Parsippany, NJ: IMS Institute for Healthcare Informatics; 2013 Oct. Google Scholar: https://scholar.google.com/scholar_lookup?title=Patient+Apps+for+Improved+Healthcare&author=M.+Aitken&author=C.+Gauntlett&publication_year=2013
