# Supplementary figures and images for: Analysis of Apps With a Medication List Functionality for Older Adults With Heart Failure Using the Mobile App Rating Scale and the IMS Institute for Healthcare Informatics Functionality Score: Evaluation Study
Source: JMIR Mhealth Uhealth. 2021 Nov 2;9(11):e30674. doi: 10.2196/30674 (PMC8596242; doi:10.2196/30674)

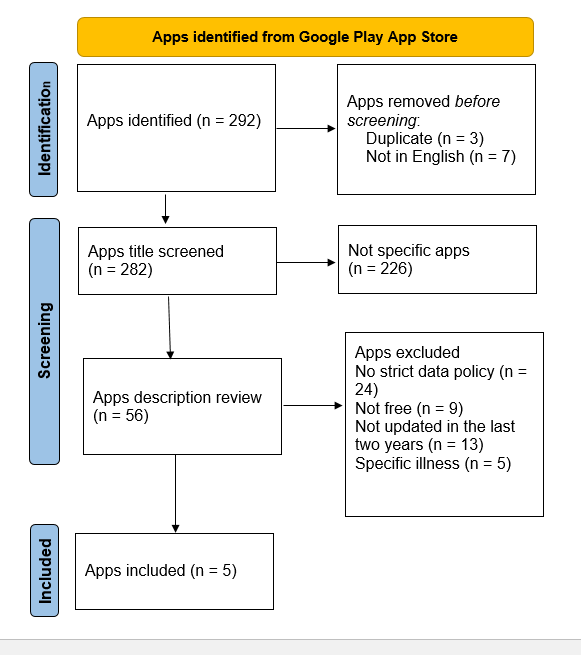


Multimedia appendix 4: App selection process flowchart Google Play app store.

Supplement: Multimedia Appendix 4 [file mhealth_v9i11e30674_app4.docx]

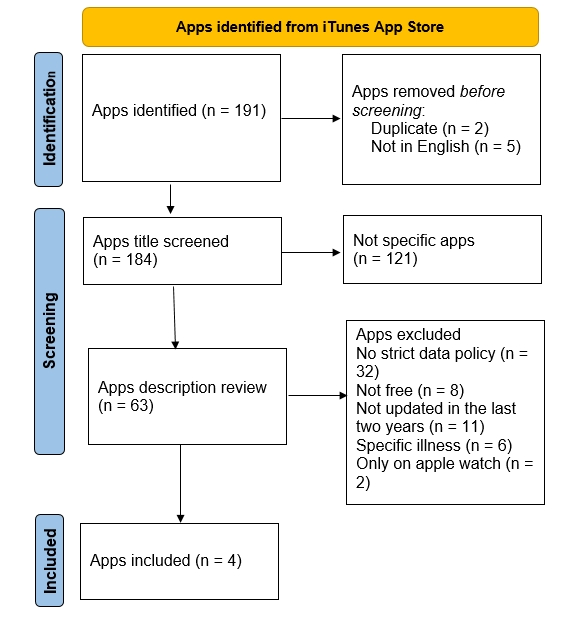


Multimedia appendix 5: App selection process iTunes app store.

Supplement: Multimedia Appendix 5 [file mhealth_v9i11e30674_app5.docx]
